# Effect of screening for type 1 diabetes on early metabolic control: the DiPiS study

**DOI:** 10.1007/s00125-018-4706-z

**Published:** 2018-08-14

**Authors:** Markus Lundgren, Berglind Jonsdottir, Helena Elding Larsson, for the DiPiS study group

**Affiliations:** 1 Unit for Pediatric Endocrinology, Department of Clinical Sciences Malmö, Lund University, Jan Waldenströms gata 35, S-205 02 Malmö, Sweden; 2 Department of Pediatrics, Kristianstad Central Hospital, Kristianstad, Sweden; 3 Pediatric Endocrinology and Gastroenterology, Skåne University hospital, Malmö, Sweden

**Keywords:** Longitudinal studies, Metabolic control, Screening, Type 1 diabetes

## Abstract

**Aims/hypothesis:**

It has been shown that children previously enrolled in follow-up studies have better glycaemic control during the early period after diabetes diagnosis. The aim of this study was to analyse glycaemic control over a longer period, past the period of partial remission, after diagnosis in children followed before diagnosis in the Swedish Diabetes Prediction in Skåne (DiPiS) study compared with children of equal age not enrolled in pre-diabetes follow-up, receiving equivalent diabetes care.

**Methods:**

HbA_1c_ from diagnosis and for the following 5 years, as well as differences in insulin dosage, BMI, pump use, partial remission according to insulin dose-adjusted HbA_1c_ and baseline demographics were compared between children who were enrolled in follow-up and had received information on diabetes risk (*n* = 51) and children not enrolled in follow-up (*n* = 78).

**Results:**

The group followed before diagnosis had a higher proportion of first-degree relatives (FDRs) with diabetes (28% vs 5.6%; *p* = 0.001) and a higher proportion of participants with mothers born in Sweden (100% vs 89%; *p* = 0.02). No significant differences in total daily insulin dose, pump use or other baseline sociodemographic factors were detected between the groups. Median HbA_1c_ at diagnosis and at 1, 2, 3, 4 and 5 years after diabetes diagnosis was significantly lower in children followed before diagnosis (all *p* < 0.05), and was not related to FDR status.

**Conclusions/interpretation:**

Compared with controls not previously enrolled in follow-up, our study shows that children enrolled in longitudinal follow-up before the diagnosis of diabetes have better glycaemic control, measured by HbA_1c_, up to 5 years after diagnosis and during the initial period of partial remission. Improved glycaemic control in the initial years of living with type 1 diabetes could affect long-term outcome and complications and might also improve study enrolment in future longitudinal studies.

**Electronic supplementary material:**

The online version of this article (10.1007/s00125-018-4706-z) contains peer-reviewed but unedited supplementary material, which is available to authorised users.

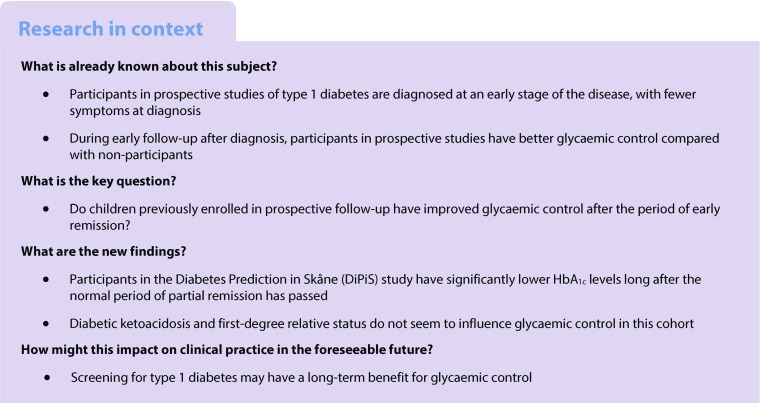



## Introduction

Several longitudinal prospective studies have followed children at increased risk for type 1 diabetes. A secondary objective in some studies has been to investigate whether study participation influenced clinical status at, and after, diagnosis. Children enrolled in follow-up studies have been reported to have a lower frequency of diabetic ketoacidosis (DKA) and other metabolic abnormalities at diagnosis [1, 2] as well as a milder clinical course after diagnosis [3]. However, the long-term effects of these findings are not clear.

It has been proposed that early diagnosis and initiation of insulin treatment could have a beneficial effect on preservation of beta cell mass [4]. Recent data contradict this and report a similar pattern of C-peptide decline even if diabetes is diagnosed very early [5]. Despite this, children who have been enrolled in prospective follow-up tend to have improved glycaemic control compared with the general population [3].

The Diabetes Prediction in Skåne (DiPiS) study is a prospective, longitudinal study of children at increased risk for type 1 diabetes. We have previously described improved metabolic status at diagnosis and the 2 years after diagnosis for study participants [6]. The aim of this study was to examine whether the positive effect on metabolic control remains after the cohort has reached 5 years after diabetes diagnosis.

## Methods

The DiPiS study is a prospective, longitudinal study on diabetes prediction in the southern part of Sweden where 38,683 children were screened for type 1 diabetes risk between September 2000 and August 2004. When the child was 2 months of age, parents were invited to participate in the study and asked to answer a questionnaire regarding pregnancy, perinatal and socioeconomic factors. When the child was 2 years old, parents were asked if they would like to be informed of the type 1 diabetes risk of their child. If they agreed, risk information was then sent to parents.

Participants were screened yearly for islet autoantibodies and were asked to complete a questionnaire. Children who developed two or more islet autoantibodies were offered follow-up every 3 months with autoantibody sampling, HbA_1c_, random plasma glucose and yearly oral glucose tolerance tests [6]. Children in follow-up were informed of risk after each blood sampling. The regional ethics review board in Lund, Sweden, approved the study. All parents gave informed consent to participate.

### Study group definition

**Fig. 1:**
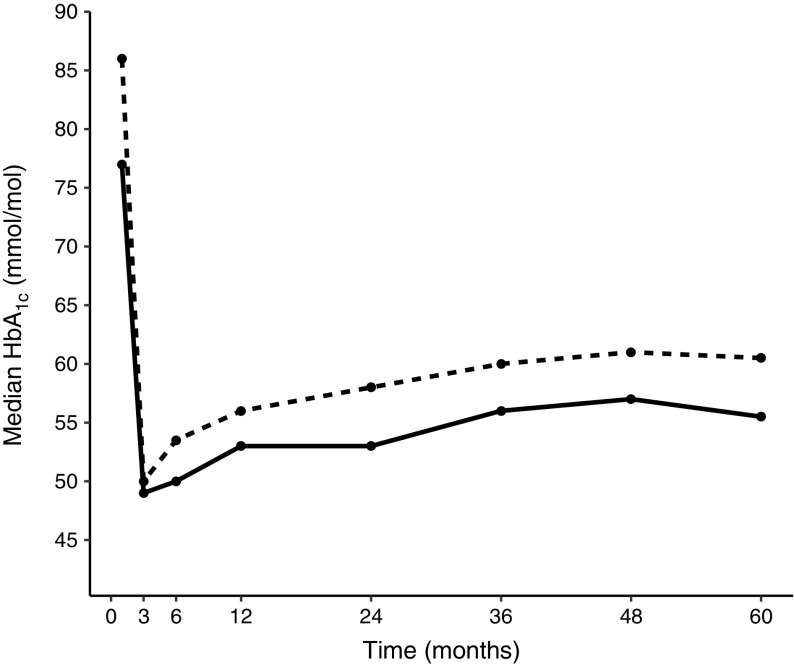
HbA_1c_ levels at diagnosis and the 5 years following diagnosis. Solid line, FU group; dotted line, NFU group

For this study, the 143 children born during 2000–2004 in the Skåne region and diagnosed with type 1 diabetes before 31 July 2013 were selected, 14 of whom were excluded because they were diagnosed before 2 years of age. This study compares the group of 51 children who had some degree of follow-up before diabetes diagnosis and who had received information on type 1 diabetes risk (follow-up group [FU]) with the 78 children who had declined to participate and had not received any information on type 1 diabetes risk (no follow-up group [NFU]) (electronic supplementary material [ESM] Fig. 1). Baseline demographics and status at diagnosis have been described previously [6].

### Baseline data

Data regarding the parents’ country of birth, maternal smoking, maternal alcohol consumption during pregnancy, marital status and maternal age were collected from the study questionnaire completed when the child was 2 months of age. First-degree relative (FDR) status was retrieved from patient records and the 2 month questionnaire. Unfortunately, the data on FDRs did not enable us to distinguish type 1 diabetes from other diabetes treated with insulin; hence, denoted insulin-dependent diabetes (IDDM). DKA was defined as blood pH <7.3. HLA DQ genotyping and risk stratification were performed as described previously [6].

### Follow-up data acquisition

Diagnosis of diabetes was obtained by paediatrician report and from the diabetes incidence register of the Better Diabetes Diagnosis study (BDD) [7]. Following diagnosis, children received care from one of the region’s six paediatric diabetes centres. No study visits were performed after diagnosis. Follow-up data were recorded at 3, 6, 12 and 24 months (±1 month), 3 and 4 years (±3 months), and 5 years (±6 months) post-diagnosis. HbA_1c_ was analysed using a DCA Vantage+ (Bayer, Tarrytown, NY, USA) or Alere Afinion (Alere, Waltham, MA, USA) analyser, both of which were calibrated by the same central laboratory. C-peptide measurements were not determined for participants after diagnosis. Insulin dose–adjusted HbA_1c_ (IDAA_1c_) was defined as HbA_1c_ (in %) + [4 × insulin dose (in U kg^−1^ day^−1^)], where partial remission was defined as an IDAA_1c_ of ≤9 [8].

### Statistical analysis

Differences between continuous variables were calculated using the Mann–Whitney *U* test and between categorical variables using Pearson’s χ^2^ and Fisher’s exact test. A *p* value <0.05 was considered significant. Data were analysed using R version 3.44 (https://www.r-project.org).

## Results

### Baseline demographics

A total of 129 children diagnosed with diabetes were included in the analysis, 51 in the FU group and 78 in the NFU group. The mean age at diagnosis was 6.8 ± 2.9 (mean ± SD) years in the FU group and 6.5 ± 2.6 years in the NFU group (ESM Table 1). The FU group had a higher frequency of FDRs with IDDM compared with the NFU group (*n* = 14 [28%] vs *n* = 4 [5.6%], respectively, *p* = 0.001). The FU group had a higher proportion of mothers born in Sweden (*n* = 50 [100%]) compared with the NFU group (*n* = 39 [88.6%], *p* = 0.02). Hereditary and sociodemographic factors are presented in ESM Table 1.

### Insulin use and partial remission

No significant differences in total daily insulin dose were observed between the two groups during follow-up (all *p* > 0.05) (Table 1). The use of insulin pumps (continuous subcutaneous insulin infusion; CSII) during follow-up and the proportion of children using CSII did not differ between the two groups at any time point (ESM Table 1). The cumulative number of years on CSII also did not differ (*p* = 0.73) (ESM Table 1). Frequency of partial remission, defined as IDAA_1c_ ≤9, was the same in both groups at all time-points except at 3 months after diagnosis (ESM Table 1).

**Table 1:** HbA_1c_ and total daily insulin dose during the first 5 years after diabetes diagnosis

Variable	FU group			NFU group			*p*
	*n*	Median (IQR)	Median (IQR)	*n*	Median (IQR)	Median (IQR)	
HbA_1c_		IFCC (mmol/mol)	NGSP (%)		IFCC (mmol/mol)	NGSP (%)	
Diagnosis	51	77 (27)	9.2 (4.6)	77	86 (23.5)	10.0 (4.3)	0.006
3 months	48	49 (11.5)	6.6 (3.2)	75	50 (14)	6.7 (3.4)	0.261
6 months	51	50 (15)	6.7 (3.5)	76	53.5 (17)	7.0 (3.7)	0.479
1 year	51	53 (9)	7.0 (3.0)	78	56 (15)	7.3 (3.5)	0.012
2 years	51	53(12)	7.0 (3.2)	77	58 (15)	7.5 (3.5)	0.002
3 years	51	56 (9.8)	7.3 (3.0)	73	60 (10.5)	7.6 (3.1)	0.014
4 years	50	57 (11)	7.4 (3.2)	75	61 (8.5)	7.7 (2.9)	0.001
5 years	46	55.5 (12)	7.2 (3.2)	74	60.5 (12)	7.7 (3.2)	0.012
TDD (U kg^−1^ day^−1^)							
3 months	50	0.47 (0.287)	–	78	0.52 (0.265)	–	0.056
6 months	50	0.60 (0.35)	–	77	0.62 (0.33)	–	0.435
1 year	50	0.74 (0.36)	–	77	0.75 (0.31)	–	0.733
2 years	50	0.80 (0.41)	–	76	0.84 (0.35)	–	0.655
3 years	43	0.83 (0.39)	–	64	0.83 (0.38)	–	0.384
4 years	44	0.92 (0.41)	–	67	0.93 (0.41)	–	0.842
5 years	37	0.85 (0.40)	–	63	0.92 (0.55)	–	0.157

Differences between groups were calculated using the Mann–Whitney *U* test

TDD, total daily insulin dose

### HbA_1c_ after diagnosis

Compared with the NFU group, HbA_1c_ levels at diabetes diagnosis were significantly lower in the FU group (*p* = 0.006), as well as at 1 year (*p* = 0.012), 2 years (*p* = 0.002), 3 years (*p* = 0.014), 4 years (*p* = 0.001), and 5 years (*p* = 0.012) after the diagnosis (Table 1). The median HbA_1c_ during the total 5 year follow-up was lower for the FU group (53.3 mmol/mol, interquartile range [IQR] 7.5) than for the NFU group (57.7 mmol/mol, [IQR 8.6]; *p* = 0.001). No differences were observed at 3 and 6 months after diagnosis (*p* = 0.26 and *p* = 0.48, respectively) (Fig. 1, Table 1). A separate analysis excluding all participants with DKA at diagnosis, was concordant with the full cohort analysis (ESM Table 2). In addition, an analysis excluding all participants with an FDR with IDDM was also consistent with the total cohort analysis (ESM Table 3).

## Discussion

In this study, we found that children previously enrolled in longitudinal follow-up have better metabolic control at diagnosis of diabetes, and that this positive effect on HbA_1c_ in this cohort remains for at least 5 years. In this study we compared children from the general population with a group previously enrolled in a prospective study on diabetes prediction; the clinical follow-up after diagnosis did not differ between the groups.

Several longitudinal studies have previously reported that children, followed for their increased type 1 diabetes risk, are diagnosed at an early stage of the disease with less morbidity and better initial glycaemic control [1, 3]. Additionally, The Environmental Determinants of Diabetes in the Young (TEDDY) study has reported both lower frequency of DKA and symptoms at diagnosis, as well as higher levels of C-peptide, for children previously enrolled in their study [9]. Recent data also show that families of children who were enrolled in the TEDDY study have better diabetes-related quality of life and lower parental parenting stress after diabetes diagnosis compared with children from the general population [10]. That an early diagnosis would lead to higher levels of remaining C-peptide has been proposed as an important factor. However, it is surprising that this effect would last as long as 5 years [5]. The FU group had a significantly lower frequency of DKA at diagnosis than the NFU group, and this has been attributed to higher levels of residual C-peptide [11]. In this cohort, no differences in remission were detected when analysing IDAA_1c_ or excluding participants with DKA at diagnosis.

Weaknesses of this analysis include measurement of a limited number of variables during clinical care, including a lack of C-peptide and of updated psychosocial and psychological data. Another limitation is that two different analysers (the DCA Vantage+ and Alere Infineon) were used to determine HbA_1c_ measurements, although they were calibrated by the same central laboratory. Strengths of the current study include the wide-scale screening of the DiPiS study, follow-up after diagnosis with similar diabetes care, data retrieval from patient records and incidence data from the BDD study with excellent coverage.

In conclusion, our study provides evidence that children enrolled in follow-up in the DiPiS study before diabetes diagnosis have better glycaemic control after diagnosis than children who did not participate in the study, and that this effect remains for at least 5 years after diagnosis. This improved glycaemic control could improve long-term outcome and reduce the risk of complications.

## Electronic supplementary material

ESM (PDF 258 kb)

## Data Availability

The datasets generated during and/or analysed during the current study are available from the corresponding author on reasonable request.

## Abbreviations

BDD: Better diabetes diagnosis study
CSII: Continuous subcutaneous insulin infusion
DiPiS: Diabetes Prediction in Skåne
DKA: Diabetes ketoacidosis
FDR: First-degree relative
FU: Follow-up group
IDAA_1c_: Insulin dose-adjusted HbA_1c_
IDDM: Insulin-dependent diabetes mellitus
NFU: No follow-up group
TEDDY: The Environmental Determinants of Diabetes in the Young
