# Supplementary material for: Effect of screening for type 1 diabetes on early metabolic control: the DiPiS study
Source: Diabetologia. 2018 Aug 14;62(1):53–7. doi: 10.1007/s00125-018-4706-z (PMC6290658; doi:10.1007/s00125-018-4706-z)

ESM table 1. Demographics of the Follow-up and No-Follow-up groups. [6]

	Study group in DiPiS				
	FU		NFU		*p*
n	51		78		
Age at diagnosis, mean (sd)	6.78	(2.87)	6.46	(2.62)	0.52
Girls, n (%)	28	(54.9)	38	(48.7)	0.61
Asymptomatic at diagnosis, n (%)[a]	9	(17.6)	2	(2.6)	0.007
DKA at diagnosis, n (%)[b]	1	(2.0)	14	(18)	0.015
HLA risk group, n (%)[c]					0.016
DQ 2/8	20	(39.2)	34	(43.6)	
DQ 8/8 or 8/X	23	(45.1)	18	(23.1)	
DQ 2/2 or 2/X	7	(13.7)	15	(19.2)	
DQ X/X	1	(2.0)	11	(14.1)	
First-degree relative, n (%)	14	(28.0)	4	(5.6)	0.001
Maternal age, mean (sd)	31.33	(4.76)	30.27	(4.95)	0.23
Smoked during pregnancy, n (%)	3	(6.0)	7	(15.9)	0.22
Alcohol use during pregnancy, n (%)	9	(18.0)	7	(15.9)	1.0
Family situation, n (%)					0.91
Live with partner	22	(44.0)	19	(45.2)	
Married	26	(52.0)	22	(52.4)	
Single	2	(4.0)	1	(2.4)	
Mother born in Sweden, n (%)	50	(100.0)	39	(88.6)	0.020
Father born in Sweden, n (%)	45	(100.0)	36	(92.3)	0.096
CSII use at 3 months, n (%)	0	(0.0)	0	(0.0)	1.0
CSII use at 6 months, n (%)	0	(0.0)	1	(1.4)	1.0
CSII use at 12 months, n (%)	6	(11.8)	9	(12.0)	1.0
CSII use at 24 months, n (%)	11	(21.6)	17	(23.0)	1.0
CSII use at 36 months, n (%)	17	(33.3)	25	(33.8)	1.0
CSII use at 48 months, n (%)	22	(44.0)	28	(37.8)	0.62
CSII use at 60 months, n (%)	28	(57.1)	34	(45.9)	0.30
CSII during follow-up, n (%)	30	(60.0)	37	(49.3)	0.32
Years on CSII, mean (sd)	1.65	(1.81)	1.53	(1.89)	0.73
BMI Sd at 12 months follow-up, mean (sd)	0.44	(1.06)	0.38	(1.10)	0.75
BMI Sd at 60 months follow-up, mean (sd)	0.80	(0.96)	0.62	(1.01)	0.32
BMI Sd change during follow-up, mean (sd)	0.31	(0.78)	0.25	(0.74)	0.65
Remission at 3 months, n (%)[d]	14	(29.8)	32	(42.7)	0.22
Remission at 6 months, n (%)	24	(48.0)	44	(57.9)	0.36
Remission at 12 months, n (%)	38	(76.0)	62	(80.5)	0.70
Remission at 24 months, n (%)	44	(88.0)	70	(92.1)	0.65
Remission at 36 months, n (%)	43	(100.0)	61	(95.3)	0.40
Remission at 48 months, n (%)	40	(90.9)	66	(98.5)	0.16
Remission at 60 months, n (%)	34	(91.9)	60	(95.2)	0.81

All percentages in columns three and five are valid percentages, ignoring subjects with missing data.
a: No polydipsia, polyuria, weight loss or DKA; b: pH <7.3 at diagnosis; c: X is neither 2 nor 8; d: Remission according to insulin dose adjusted HbA1c ≤ 9.0.

ESM Table 2. HbA1c during the first five years after diabetes diagnosis, excluding participants with ketoacidosis at diagnosis

	Follow-up (FU)			No Follow-up (NFU)			
	n	IFCC Median mmol/mol (IQR)	NGSP Median % (IQR)	n	IFCC Median mmol/mol(IQR)	NGSP Median % (IQR)	*p*
Diagnosis	51	76.0 (26)	9.10 (2.0)	77	83.5 (22)	9.79 (2.0)	0.044
3 Months	48	49.0 (10)	6.63 (0.9)	75	50.0 (11)	6.73 (1.0)	0.064
6 Months	51	51.0 (14)	6.82 (1.3)	76	54.0 (15)	7.09 (1.3)	0.223
1 year	51	53.0 (8.0)	7.00 (0.7)	78	57.0 (14)	7.37 (1.3)	0.003
2 years	51	53.0 (12)	7.00 (1.1)	77	59.0 (14)	7.55 (1.2)	0.001
3 years	51	56.0 (10)	7.27 (0.9)	73	60.0 (10)	7.64 (0.9)	0.006
4 years	50	57.0 (12)	7.37 (1.1)	75	61.0 (8.0)	7.73 (0.7)	0.001
5 years	46	56.5 (13)	7.23 (1.2)	74	61.0 (12)	7.73 (1.1)	0.011

ESM Table 3. HbA1c during the first five years after diabetes diagnosis, excluding participants with first degree relatives with insulin dependent diabetes mellitus

	Follow-up (FU)			No Follow-up (NFU)			
	n	IFCC Median mmol/mol (IQR)	NGSP Median % (IQR)	n	IFCC Median mmol/mol (IQR)	NGSP Median % (IQR)	*p*
Diagnosis	36	80.5 (25)	9.52 (2.3)	66	86.0 (23)	10.02 (2.1)	0.020
3 Months	34	49.0 (7.8)	6.63 (0.7)	65	49.0 (12)	6.63 (1.1)	0.533
6 Months	36	49.5 (12)	6.68 (1.1)	65	53.0 (16)	7.00 (1.5)	0.694
1 year	36	52.0 (16)	6.91 (0.6)	67	56.0 (14)	7.27 (1.2)	0.009
2 years	36	52.5 (14)	6.95 (1.2)	66	57.0 (8.8)	7.37 (1.4)	0.003
3 years	36	54.5 (8.8)	7.14 (0.8)	62	59.0 (9.0)	7.55 (0.8)	0.007
4 years	35	55.0 (11)	7.18 (1.0)	64	60.5 (12)	7.69 (1.1)	<0.001
5 years	32	55.0 (13)	7.18 (1.2)	63	60.0 (14)	7.64 (1.3)	0.008

ESM Figure 1: Inclusion in the present study

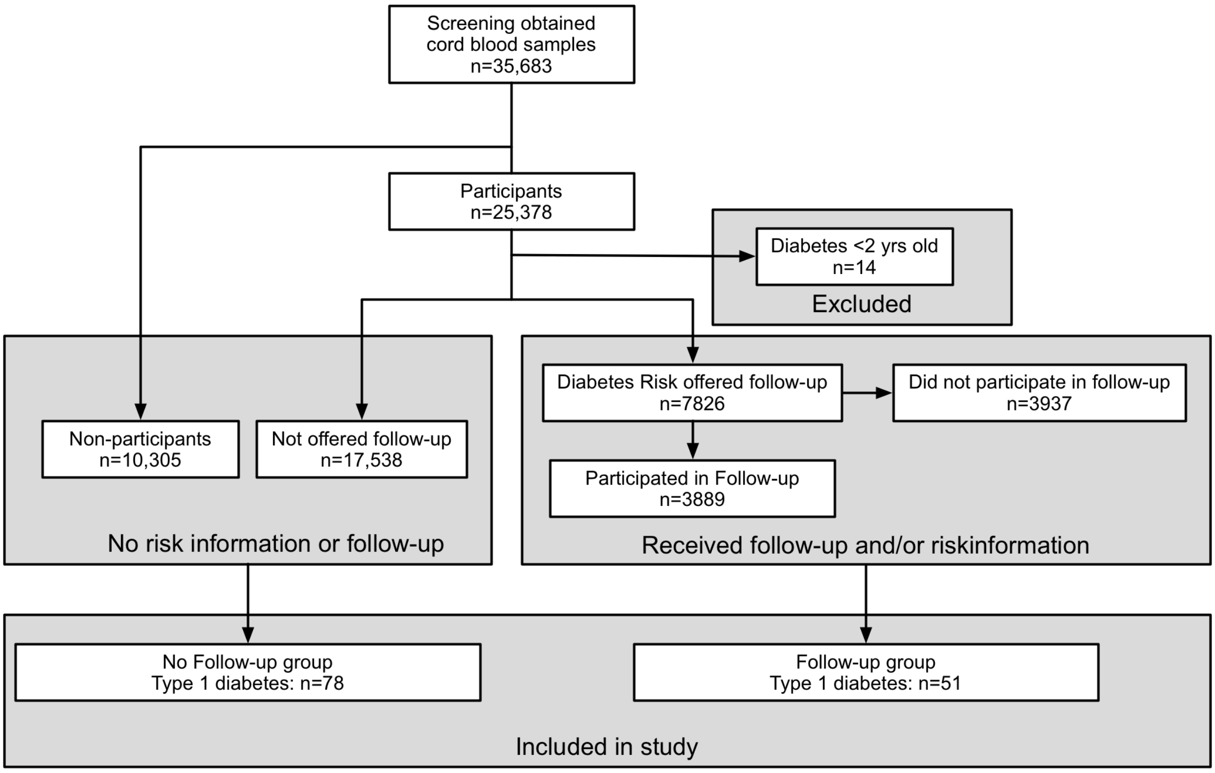

Supplement: Supplementary file 1 — (PDF 258 kb) [file 125_2018_4706_MOESM1_ESM.pdf]
